# Supplementary material for: Intermittent fasting induces rapid hepatocyte proliferation to restore the hepatostat in the mouse liver
Source: eLife. 2023 Jan 31;12:e82311. doi: 10.7554/eLife.82311 (PMC9889086; doi:10.7554/eLife.82311)
Supplement: Supplementary file 1. [file elife-82311-supp1.docx]

|  | **FC** | **log2(FC)** | **p.ajusted** | **-log10(p)** |
| --- | --- | --- | --- | --- |
| **OPHTHALMIC ACID** | 0.18115 | -2.4647 | 0.012447 | 1.9049 |
| **Methacholine cation** | 0.19225 | -2.3789 | 0.012447 | 1.9049 |
| **Putrescine** | 4.3578 | 2.1236 | 0.012447 | 1.9049 |
| **7-Oxocholic acid** | 0.0021904 | -8.8346 | 0.016196 | 1.7906 |
| **Taurine** | 4.5445 | 2.1841 | 0.016196 | 1.7906 |
| **Ser-Arg** | 0.3967 | -1.3339 | 0.016196 | 1.7906 |
| **O-Phosphocolamine** | 0.46584 | -1.1021 | 0.016196 | 1.7906 |
| **Equol** | 8.3938 | 3.0693 | 0.02186 | 1.6603 |
| **PC 32:1** | 2.5894 | 1.3726 | 0.022122 | 1.6552 |
| **N-Methylglutamic acid** | 0.26765 | -1.9016 | 0.022723 | 1.6435 |
| **N-omega-Acetylhistamine** | 0.18058 | -2.4693 | 0.023334 | 1.632 |
| **Thiamine monophosphate** | 0.34742 | -1.5252 | 0.023334 | 1.632 |
| **PG 40:8** | 0.31159 | -1.6823 | 0.024099 | 1.618 |
| **gamma-Glutamylleucine** | 0.40523 | -1.3032 | 0.025438 | 1.5945 |
| **Thr-His** | 0.26486 | -1.9167 | 0.025814 | 1.5881 |
| **1-Methyl-L-histidine** | 0.36235 | -1.4645 | 0.025814 | 1.5881 |
| **Gln-Ala** | 0.17035 | -2.5535 | 0.029313 | 1.5329 |
| **3-Oxocholic acid** | 0.022006 | -5.5059 | 0.033364 | 1.4767 |
| **Ser-Ser** | 0.31113 | -1.6844 | 0.033364 | 1.4767 |
| **Carnosine** | 0.32891 | -1.6042 | 0.033364 | 1.4767 |
| **1-Methylhistamine** | 0.37256 | -1.4245 | 0.033364 | 1.4767 |
| **His-Ala** | 0.37309 | -1.4224 | 0.033364 | 1.4767 |
| **PE 40:8** | 0.40471 | -1.305 | 0.033364 | 1.4767 |
| **Cytosine** | 2.4509 | 1.2933 | 0.033364 | 1.4767 |
| **Arg-Tyr** | 0.42324 | -1.2405 | 0.033364 | 1.4767 |
| **Phenylacetylglycine** | 0.47092 | -1.0865 | 0.033364 | 1.4767 |
| **N-ACETYL-DL-METHIONINE** | 0.28691 | -1.8013 | 0.035354 | 1.4516 |
| **Ser-Gly** | 0.31669 | -1.6588 | 0.037711 | 1.4235 |
| **3-Methylhistidine** | 0.39726 | -1.3318 | 0.040853 | 1.3888 |
| **1-(1Z-Octadecenyl)-sn-glycero-3-phosphocholine** | 0.48902 | -1.032 | 0.04227 | 1.374 |
| **2-Methylbutyryl-L-carnitine** | 2.4575 | 1.2972 | 0.042326 | 1.3734 |
| **lysoPE 16:1** | 2.0003 | 1.0002 | 0.042326 | 1.3734 |
| **Ser-Thr** | 0.37912 | -1.3993 | 0.043832 | 1.3582 |
| **Ser-Asn** | 0.25368 | -1.9789 | 0.044216 | 1.3544 |
| **Cholic acid** | 0.017016 | -5.877 | 0.046539 | 1.3322 |
